# *Platycodon grandiflorus* Polysaccharide Attenuates Inflammation by Inhibiting NLRP3 Inflammasome Activation via the ROS/NEK7 Pathway

**DOI:** 10.3390/molecules31132271

**Published:** 2026-06-29

**Authors:** Meiyun Lv, Yue Yu, Linjue Li, Yang Liu, Zhaolong Li, Xiaoran Zhang, Xinyi Dai, Pimiao Zheng, Jianzhu Liu, Xiaona Zhao

**Affiliations:** 1 College of Veterinary Medicine, Shandong Agricultural University, Tai’an 271018, China; lvmeiyun2022@163.com (M.L.); 15255353559@139.com (Y.Y.); cheyouyoua@163.com (L.L.); yangliusdau163@163.com (Y.L.); m13513034321@163.com (Z.L.); 15563922690@163.com (X.Z.); 15269663558@163.com (X.D.); zpmiao00@163.com (P.Z.); 2 Shandong Provincial Key Laboratory of Zoonoses, Shandong Agricultural University, Tai’an 271018, China

**Keywords:** *Platycodon grandiflorus* polysaccharide, NLRP3 inflammasome, NEK7

## Abstract

Dysregulated activation of the NLRP3 inflammasome is a key driver in the pathogenesis of numerous inflammatory disorders. This study aimed to evaluate the protective effect of *Platycodon grandiflorus* polysaccharide (PGPS_t_) against NLRP3-inflammasome-mediated inflammation and elucidate its underlying mechanisms. An in vitro inflammatory model was established in porcine alveolar macrophages (3D4/21) using LPS/ATP co-stimulation. The effects of PGPS_t_ were assessed by measuring inflammasome activation, intracellular reactive oxygen species (ROS) generation, and pro-inflammatory cytokine secretion. Molecular docking, alongside inhibitors (NAC, MCC950) and siRNA targeting NEK7, was employed to probe the involved mechanisms. PGPS_t_ significantly suppressed NLRP3 inflammasome assembly and activation, reduced caspase-1 cleavage, and decreased the maturation and release of IL-1β and IL-18. It exerted its inhibitory effects through dual mechanisms: scavenging intracellular ROS and directly binding to NEK7 and NLRP3 to disrupt their interaction, as supported by molecular docking. The anti-inflammatory effect was diminished upon NEK7 knockdown. In conclusion, PGPS_t_ is an effective natural inhibitor of the NLRP3 inflammasome, functioning through ROS clearance and direct interference with the NLRP3–NEK7 interaction. These findings propose PGPS_t_ as a promising therapeutic candidate and further validate NEK7 as a potential target for treating NLRP3-driven inflammatory diseases.

## 1. Introduction

*Platycodon grandiflorus* (Jacq.) A. DC. (PG) is a well-documented medicinal-food homologous plant in China, valued for both culinary and therapeutic applications [1]. Polysaccharides isolated from its roots (PGPS_t_) represent critical bioactive constituents, demonstrating a range of pharmacological effects including immunomodulatory, anti-inflammatory, antioxidant, and antiviral activities [2]. In our prior studies, we found that PGPS_t_ alleviates Cr (VI)-induced autophagy by reducing ROS levels and restoring mitochondrial membrane potential (MMP) [3]. Other evidence also supports its potential for treating acute liver injury by reducing oxidative stress and pro-inflammatory cytokines [4], underscoring its broad therapeutic value.

Numerous inflammatory and autoimmune disorders, including bacterial infections, atherosclerosis, type II diabetes, obesity, and rheumatoid arthritis, are driven by inflammation [5]. Moderating inflammatory responses effectively helps alleviate immunopathological changes and immune-mediated tissue damage. Macrophages are key initiators of the inflammatory response and play a central role in the pathogenesis of numerous inflammatory diseases, primarily by secreting a range of pro-inflammatory mediators and cytokines [6]. Inflammasomes, multiprotein complexes, are vital in inflammation and immunity. They are multimeric protein structures that control the inflammatory response and pyroptosis and are part of the host’s defense against microbes. The main components of inflammasomes are PRR (pattern recognition receptor), ASC, and caspase-1. NLRP3 has three parts: LRRs (leucine-rich repeats) for recognizing stimuli, NACHT (nucleotide-binding-and-oligomerization domain), and PYD (pyrin domain) that mediates ASC [7]. NLRP3 inflammasome formation has two stages: priming and assembly. During priming, TLRs (toll-like receptors) induce NF-κB-mediated NLRP3, pro-caspase-1, pro-IL-1β, and pro-IL-18 due to PAMP (pathogen-related molecular model) and DAMP (danger-related molecular pattern). During the assembly process, activated NLRP3 interacts with ASC and pro-caspase-1 to form a large multiprotein complex. This leads to the maturation and secretion of interleukins like IL-1β and IL-18 and other pro-inflammatory soluble mediators [8]. The NLRP3 inflammasome, when over-activated, can induce apoptosis and participate in disease progression by producing excess inflammatory cytokines. Accumulating studies have demonstrated that excessive activation of the NLRP3 inflammasome is involved in various inflammatory, metabolic, autoimmune, and neoplastic diseases, as well as oncological disorders. Currently, there is a growing body of research targeting the NLRP3 inflammasome to treat related diseases by modulating the inflammatory response.

ROS are unstable, highly reactive molecules, which are formed primarily by oxygen reduction during oxidative phosphorylation in mitochondria [9]. Excessive ROS generation and/or failure of the anti-oxidant defense system can cause oxidative stress, which can lead to the destruction of cellular macromolecules [10]. ROS are thought to be a trigger for activating the NLRP3 inflammasome [11]. Many inflammatory diseases are associated with ROS, both inflammatory signaling molecules and mediators of inflammation. Long-term or chronic ROS molecules are thought to be central to the progression of inflammatory diseases. In cells where various NADPH oxidases are present, especially in specialized phagocytes and endothelial cells, ROS act as an effector center during inflammatory activation. NF-κB, a key transcription factor mediating inflammation, stress responses, and cell growth and survival, is activated by ROS [12]. Therefore, by abating the excessive synthesis of excess ROS in the body, the activation of inflammasomes can be effectively suppressed, thus alleviating the inflammatory response.

NEK7 is a centrosomal kinase needed for mitotic division [13]. The lower activity of NEK7 in natural growth may be necessary for the maintenance of homeostasis. But any disturbance in homeostasis can be associated with NEK7 dysfunction, resulting in abnormal cells, such as multinucleate cells and apoptosis [14]. NEK7 has been identified as an NLRP3-binding protein, which acts as a mediator between NLRP3 activation and the NLRP3 inflammation. Emerging research suggests that NEK7 is an essential downstream mediator for the efflux of K^+^, and it is recruited to help build inflammasomes upstream in response to NLRP3 activation [15]. The NLRP3/ASC complex also contains NEK7, and the interaction of NEK7 with NLRP3 is necessary for the formation of the NLRP3 complex, which is an upstream step in the assembly of the ASC complex. [16]. These results suggest that NEK7 may not only contribute to the release of intracellular K^+^, but also may be an essential protein in the induction of inflammation. Therefore, it can be used as a means to suppress the inflammatory response by acting on NEK7.

Building on the established role of NLRP3 in driving inflammatory pathologies, we therefore postulated that PGPS_t_ mediates its ability to reduce inflammation by specifically focusing on the NLRP3 inflammasome axis in macrophages. We specifically postulated that PGPS_t_ would suppress the NLRP3 inflammasome assembly and stimulation through mitigating the upstream reactive oxygen species (ROS) burst and, more importantly, by directly interfering with the critical NLRP3–NEK7 interaction. Our findings establish PGPS_t_ as a potent, natural-product-derived inhibitor of the NLRP3 inflammasome pathway. Specifically, we demonstrate that its inhibitory action is achieved by disrupting the NLRP3–NEK7 interaction, a crucial checkpoint for inflammasome assembly. Furthermore, supported by molecular docking predictions, our work uniquely highlights NEK7 not only as a key mediator of PGPS_t_’s action but also as a potentially effective new treatment target for inflammatory disorders caused by NLRP3.

## 2. Results

### 2.1. Determination of Optimal LPS Concentration and Duration for Standardized Inflammatory Stimulation

**Figure 1:**
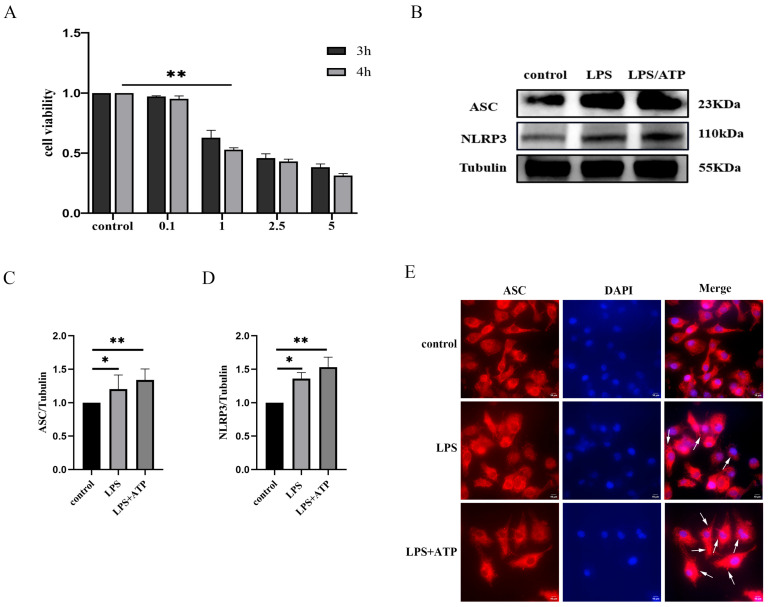
Optimization of LPS stimulation and NLRP3 inflammasome activation by LPS plus ATP in 3D4/21 cells. (**A**) Cell viability, measured by CCK-8 assay, after treatment with the indicated concentrations of LPS (0.1–5 μg/mL) for 3 or 4 h. (**B**) Representative Western blots showing the protein levels of ASC and NLRP3. Cells were treated with LPS (1 μg/mL, 4 h) alone or followed by ATP (5 mM, 1 h; LPS/ATP group). (**C**,**D**) Densitometric quantification of ASC (**C**) and NLRP3 (**D**) protein levels from Western blot analyses, normalized to a loading control. (**E**) Representative immunofluorescence images showing ASC distribution (red). Nuclei were counterstained with Hoechst 33342 (blue). Arrows indicate ASC specks. The white arrows indicate the formation of ASC specks, a hallmark of inflammasome activation. Data are presented as mean ± SD (*n* = 3). * *p* < 0.05, ** *p* < 0.01 versus the control group.

For the purpose of constructing an in vitro model of inflammation in cultured cells, the optimal LPS stimulation conditions were first determined using 3D4/21 cells. We treated the cells with a series of LPS concentrations (0.1, 1.0, 2.5, and 5.0 μg/mL) to screen for the optimal stimulation dose, with incubations lasting 3 or 4 h, with cellular viability measured via CCK-8 cell viability assay to evaluate inflammatory injury. The results showed that LPS induced a dose- and time-dependent reduction in cell viability. Specifically, treatment with 1.0 μg/mL LPS for 4 h led to a 50.6% inhibition rate in comparison with the control group (*p* < 0.01; Figure 1A). On the basis of these observations, subsequent experiments adopted stimulation with 1.0 μg/mL LPS for 4 h to induce inflammation.

### 2.2. LPS and ATP Co-Stimulate 3D4/21 Cells to Trigger Inflammation

The combination of LPS and ATP, established as a canonical NLRP3 inflammasome activator, effectively induces robust inflammasome activation in 3D4/21 cells. This activation manifests through significant upregulation of NLRP3 and ASC protein expression (*p* < 0.05; *p* < 0.01), as presented in Figure 1B–D. Additionally, marked increases in ASC speck formation, which represent a characteristic feature of inflammasome assembly, were observed through immunofluorescence analysis (Figure 1E). These findings confirm that LPS and ATP synergistically activate the NLRP3 inflammasome, thereby eliciting a substantial cellular reaction to inflammation in 3D4/21 cells.

### 2.3. Determination of the Optimal Anti-Inflammatory Concentration of PGPS_t_

**Figure 2:**
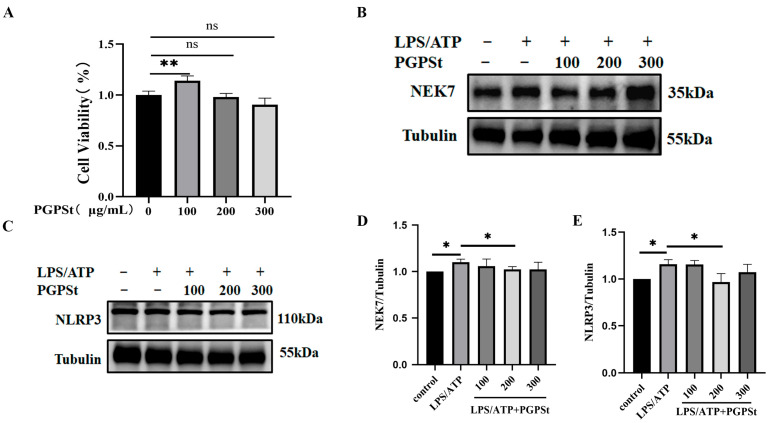
Determination of the optimal anti-inflammatory concentration of PGPS_t_. (**A**) Cell viability of 3D4/21 cells treated with PGPS_t_ alone (100, 200, or 300 μg/mL) for 24 h in the absence of LPS/ATP, as determined by CCK-8 assay. PGPS_t_ alone did not significantly affect cell viability compared with the control group (ns, *p* > 0.05). (**B**,**C**) 3D4/21 cells were pretreated with the indicated concentrations of PGPS_t_ (100, 200, or 300 μg/mL) for a specified time, followed by stimulation with LPS/ATP. Representative Western blot images show the protein levels of (**B**) NEK7 and (**C**) NLRP3. (**D**,**E**) Densitometric quantification of (**D**) NEK7 and (**E**) NLRP3 protein levels from Western blot analyses, normalized to a loading control. Data are presented as mean ± SD (*n* = 3). * *p* < 0.05, ** *p* < 0.01 versus the control or LPS/ATP group.

The cytotoxicity of PGPS_t_ was first evaluated in 3D4/21 cells using the CCK-8 assay to select a safe concentration range for subsequent experiments. As shown in Figure 2A, treatment with PGPS_t_ at 100, 200, or 300 μg/mL did not significantly affect cell viability compared with the untreated control group, indicating that PGPS_t_ was nontoxic within the tested concentration range.

To identify the optimal anti-inflammatory concentration, cells were pretreated with graded doses of PGPS_t_ (100, 200, and 300 μg/mL) prior to LPS/ATP stimulation. LPS/ATP challenge markedly upregulated the expression of NLRP3 and NEK7, two key components of the NLRP3 inflammasome, compared with the control group. Among the tested concentrations, pretreatment with 200 μg/mL PGPS_t_ most effectively suppressed LPS/ATP-induced upregulation of NLRP3 and NEK7, as confirmed by Western blot analysis (*p* < 0.05; Figure 2B–E).

Therefore, 200 μg/mL PGPS_t_ was selected for all subsequent experiments, demonstrating that PGPS_t_ can protect against LPS/ATP-induced inflammatory injury in vitro.

### 2.4. PGPS_t_ Mitigated LPS/ATP-Triggered Reactive Oxygen Species (ROS) Generation in 3D4/21 Cells

**Figure 3:**
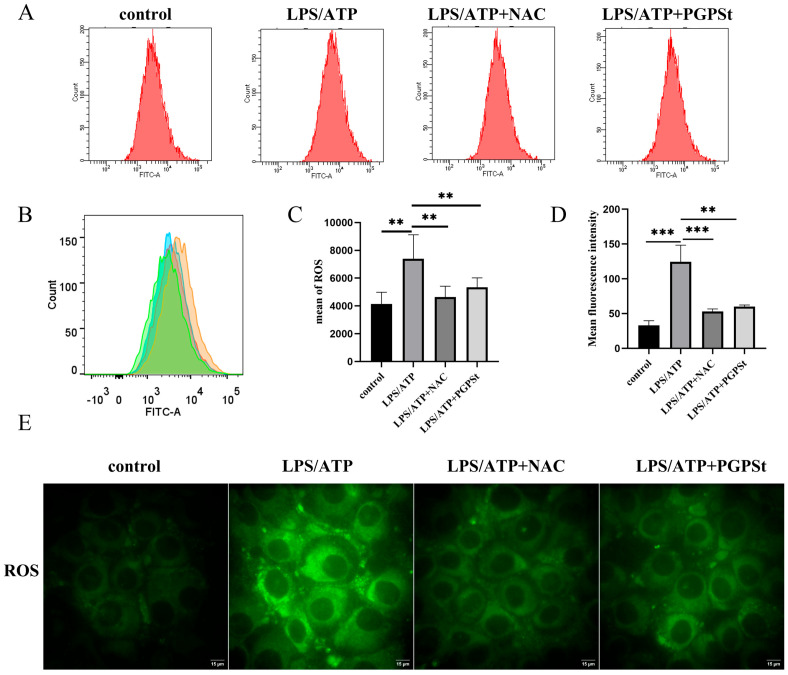
PGPS_t_ alleviates LPS/ATP-induced ROS production in 3D4/21 cells. (**A**) 3D4/21 cells were cotreated with 5 mM NAC or 200 μg/mL PGPS_t_ to analyze LPS/ATP-induced accumulation of ROS by flowcytometry. (**B**,**C**) Quantitative analysis of the mean ROS. (**D**) Quantitative analysis of the mean fluorescence intensity. (**E**) Confocal microscope images show the fluorescence intensity of ROS. Means ± SD are indicated (*n* = 3). ** indicates *p* < 0.01; *** indicates *p* < 0.001 versus the control or LPS/ATP group.

Given that ATP-induced ROS production is a key mediator of inflammation and pyroptosis, frequently derived from damaged mitochondria [17], we assessed the impact of PGPS_t_ on intracellular ROS accumulation in LPS/ATP-challenged 3D4/21 cells. Quantitative analysis by flow cytometry and qualitative observation by fluorescence microscopy, using DCFH-DA staining, showed that LPS/ATP stimulation significantly increased ROS levels. This elevation in ROS levels was strongly attenuated by concurrent treatment with 200 μg/mL PGPS_t_ or the positive control antioxidant NAC (*p* < 0.01; Figure 3A,B). Consistent with this, confocal imaging demonstrated that PGPS_t_ potently diminished the LPS/ATP-evoked green fluorescence signal (*p* < 0.01; *p* < 0.001; Figure 3C,D). Collectively, these findings confirm that PGPS_t_ potently blocks LPS/ATP-mediated ROS generation in 3D4/21 cells.

### 2.5. PGPS_t_ Inhibits the Activation of the NLRP3 Inflammasome and Cytokine Secretion Through an ROS-Dependent Pathway

**Figure 4:**
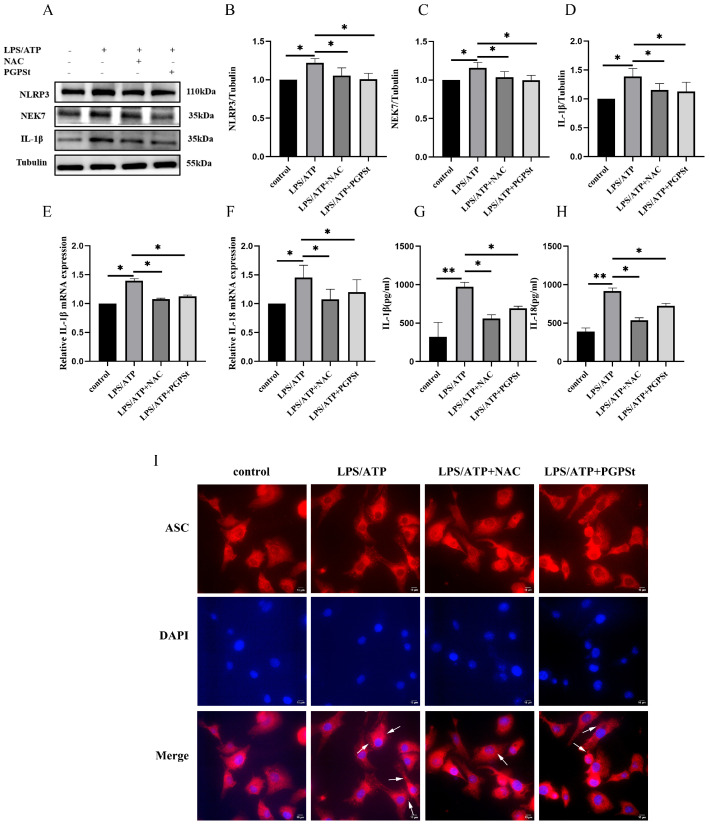
PGPS_t_ inhibits NLRP3 inflammasome activation and cytokine production in LPS/ATP-stimulated 3D4/21 cells. (**A**) Representative Western blots showing the protein levels of NLRP3, NEK7, and IL-1β in 3D4/21 cells treated as indicated. (**B**–**D**) Quantitative analysis of (**B**) NLRP3, (**C**) NEK7, and (**D**) IL-1β protein levels normalized to tubulin. (**E**,**F**) mRNA expression levels of (**E**) IL-1β and (**F**) IL-18 determined by qRT-PCR. (**G**,**H**) Secretion levels of (**G**) IL-1β and (**H**) IL-18 in cell culture supernatants measured by ELISA. (**I**) Representative immunofluorescence images showing ASC speck formation (red). Nuclei were stained with Hoechst 33342 (blue). The white arrows indicate the formation of ASC specks, a hallmark of inflammasome activation. Data are presented as mean ± SD (*n* = 3). * *p* < 0.05, ** *p* < 0.01 versus the control or LPS/ATP group.

Previous studies have established that intracellular ROS generation acts as a critical driver of NLRP3 inflammasome assembly [18]. In line with this observation, the ROS-scavenging agent N-acetylcysteine (NAC) has been shown to block caspase-1 activation and IL-1β processing. To test whether PGPS_t_ inhibits inflammasome activation via ROS reduction, 3D4/21 cells exposed to LPS/ATP were treated concurrently with 200 μg/mL PGPS_t_ or 5 mM NAC over a 5 h incubation period. Western blotting demonstrated that both PGPS_t_ and NAC significantly downregulated the protein levels of NEK7, NLRP3, and IL-1β relative to the LPS/ATP-challenged control group (*p* < 0.05; Figure 4A–D).

IL-1β and IL-18 are key pro-inflammatory cytokines that drive the initiation of immune and inflammatory responses [19]. We therefore examined whether PGPS_t_ alters the production of these cytokines and their release into the extracellular space. In line with this, the potent downregulation of LPS/ATP-induced IL-18 and IL-1β mRNA by PGPS_t_ (as shown by qPCR) matched the efficacy of NAC (*p* < 0.05; Figure 4E,F). Consistent with this, ELISA results showed that LPS/ATP stimulation significantly increased IL-1β and IL-18 secretion into the supernatant, and this elevation was significantly suppressed by either PGPS_t_ or NAC (*p* < 0.05; *p* < 0.01) (Figure 4G,H).

Activation of the NLRP3 inflammasome begins with the assembly of NLRP3, ASC, and pro-caspase-1 into a multiprotein complex, a process in which ASC speck formation marks a key step and serves as a key indicator of inflammasome activation. Immunofluorescence analysis revealed that LPS/ATP stimulation induced prominent ASC speck formation, a cellular response that was considerably diminished following PGPS_t_ administration in our experimental system (Figure 4I,J).

Collectively, these findings point to the inhibition of ROS-mediated NLRP3 inflammasome activation as the mechanism through which PGPS_t_ curtails the production and secretion of IL-1β and IL-18 in 3D4/21 cells.

### 2.6. Molecular Docking Screening Identifies D-Glucose as a High-Affinity Ligand for NLRP3 and NEK7

**Figure 5:**
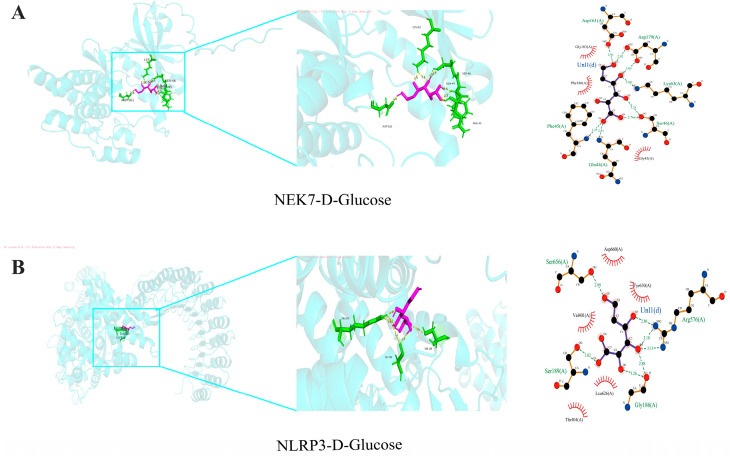
Exploring the interaction of D-Glucose (a unit of PGPSt) with NLRP3 inflammasome components. (**A**) Predicted binding mode of D-Glucose to NEK7. (**B**) Predicted binding mode of D-Glucose to NLRP3.

Molecular docking was utilized to explore the binding interactions among NLRP3 (PDB ID: H2EW08), NEK7 (PDB ID: Q9ES74), and a range of carbohydrate compounds. The docking simulations indicated that D-Glucose (PubChem CID: 5793) demonstrated the strongest binding affinity compared to the other compounds tested (Figure 5A,B).

### 2.7. PGPS_t_ Inhibits NLRP3 Inflammasome Activation and Cytokine Release Through the NEK7–NLRP3 Pathway

**Figure 6:**
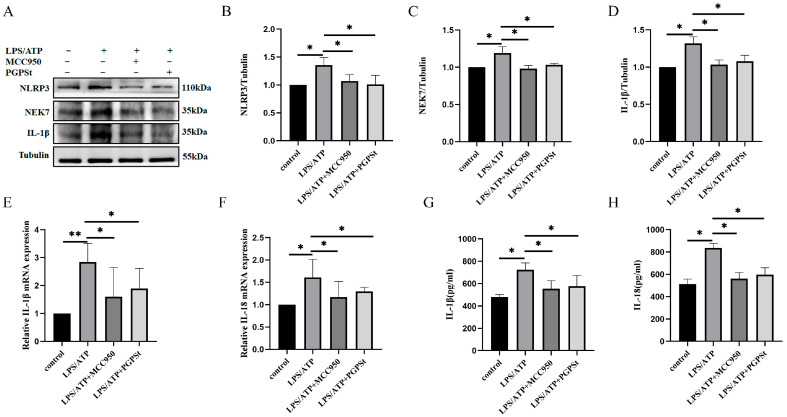
PGPS_t_ suppresses NLRP3 inflammasome activation and cytokine production via the NEK7–NLRP3 pathway. (**A**) Representative Western blots showing protein levels of NEK7, NLRP3, and IL-1β in 3D4/21 cells treated as indicated. (**B**–**D**) Quantitative analysis of (**B**) NLRP3, (**C**) NEK7, and (**D**) IL-1β protein expression normalized to tubulin. (**E**,**F**) mRNA expression levels of (**E**) IL-1β and (**F**) IL-18 measured by qRT-PCR. (**G**,**H**) Secretion levels of (**G**) IL-1β and (**H**) IL-18 in cell culture supernatants determined by ELISA. All data are presented as mean ± SD (*n* = 3). * *p* < 0.05, ** *p* < 0.01 versus the control or LPS/ATP group.

MCC950, a known NLRP3 inflammasome inhibitor, has been shown to partially suppress both NLRP3 and NEK7 [20]. In addition, ROS-dependent NEK7 phosphorylation could enhance NEK7–NLRP3 interaction and promote inflammasome activation [21]. To investigate PGPS_t_’s anti-inflammatory mechanism in more detail, we used MCC950 to inhibit the NEK7–NLRP3 pathway. Cells were exposed to 200 μg/mL PGPS_t_ or 5 μM MCC950, in combination with LPS/ATP, for 5 h. PGPS_t_ significantly suppressed the expression of the pro-inflammatory proteins NEK7, NLRP3, and IL-1β, as assessed by Western blot, with an efficacy comparable to MCC950 relative to the LPS/ATP group (*p* < 0.05; Figure 6A–D).

We next examined whether PGPS_t_ influences the mRNA expression and the subsequent secretion of IL-1β and IL-18 using qPCR and ELISA. Both PGPSt and MCC950 significantly reduced the mRNA levels of IL-1β and IL-18 relative to the LPS/ATP-stimulated group (*p* < 0.05; *p* < 0.01) (Figure 6E,F). Similarly, ELISA results indicated that the elevated secretion of IL-1β and IL-18 induced by LPS/ATP was markedly attenuated by either PGPS_t_ or MCC950 treatment (*p* < 0.05; Figure 6G,H). Thus, the anti-inflammatory action of PGPS_t_ stems from its blockade of the NEK7–NLRP3 interaction, which in turn suppresses inflammasome activation and reduces pro-inflammatory cytokine secretion.

### 2.8. PGPS_t_ Alleviates Inflammation by Downregulating NEK7, Thereby Blocking the Assembly and Activation of the NLRP3 Inflammasome

**Figure 7:**
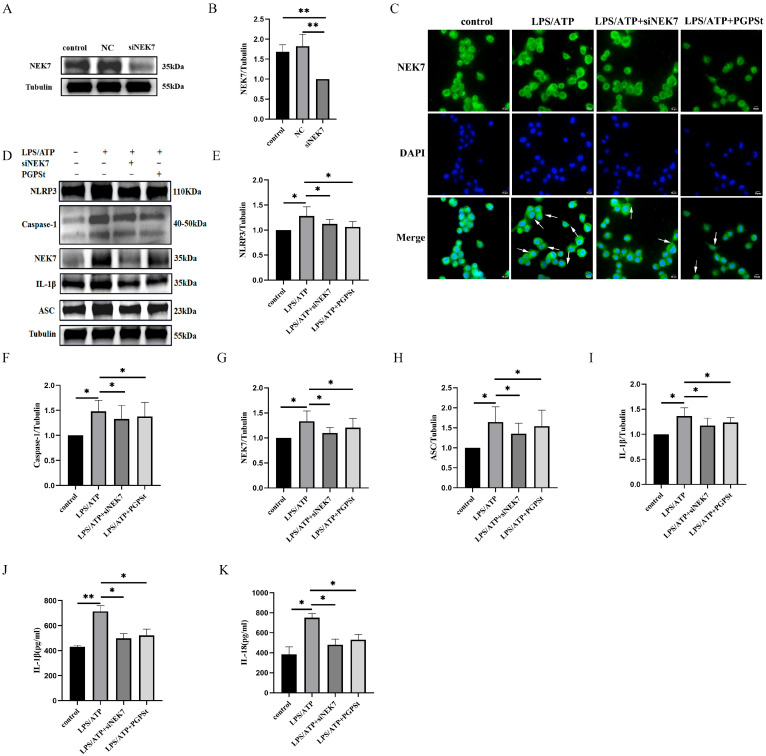
PGPS_t_ alleviates inflammation by downregulating NEK7 to suppress NLRP3 inflammasome activation. (**A**) Western blot analysis of NEK7 protein expression in cells transfected with negative control (NC) or NEK7-targeting siRNA. (**B**) Quantification of NEK7 protein levels from (**A**). (**C**) Representative immunofluorescence images showing NEK7 distribution (green) in 3D4/21 cells. Nuclei were stained with Hoechst 33342 (blue). White arrows indicate the perinuclear aggregation of NEK7, a key event in NLRP3 inflammasome activation. (**D**) Western blot analysis of NEK7, NLRP3, ASC, caspase-1, and IL-1β protein expression in cells treated as indicated. (**E**–**I**) Quantitative analysis of (**E**) NLRP3, (**F**) caspase-1, (**G**) NEK7, (**H**) ASC, and (**I**) IL-1β protein levels normalized to tubulin. (**J**,**K**) Secretion levels of (**J**) IL-1β and (**K**) IL-18 in cell culture supernatants measured by ELISA. Means ± SD are indicated (*n* = 3). * *p* < 0.05, ** *p* < 0.01 versus the control or LPS/ATP group.

PGPS_t_ inhibited the functional initiation of the NLRP3 inflammasome through interfering with the critical interaction between NLRP3 and NEK7, suggesting a potential direct targeting of NEK7 by PGPS_t_. We next aimed to clarify how NEK7 is involved in the anti-inflammatory action of PGPS_t_. To do so, NEK7 expression was knocked down using NEK7-specific siRNA. Western blot analysis confirmed a significant reduction in NEK7 protein levels in the siRNA-treated group, indicating successful silencing (*p* < 0.01; Figure 7A,B). Immunofluorescence microscopy further validated the knockdown efficiency. NEK7 expression in PGPS_t_-treated cells was markedly reduced compared to the LPS/ATP group and matched the low levels seen in NEK7-silenced cells (Figure 7C).

The effects of PGPS_t_ on NLRP3-inflammasome-related protein expression were examined. PGPS_t_ treatment resulted in downregulation of NEK7 expression, which in turn suppressed NLRP3 and ASC levels and inhibited inflammasome assembly. This reduction in NLRP3 activation impaired ASC speck formation, ultimately leading to decreased cleavage and activation of caspase-1 (*p* < 0.05; Figure 7D–I). In line with this pattern, ELISA showed that both PGPS_t_ and NEK7 siRNA significantly attenuated the LPS/ATP-induced secretion of IL-1β and IL-18 into the supernatant (*p* < 0.05, *p* < 0.01; Figure 7J,K).

We show that PGPS_t_ exerts its anti-inflammatory effect by targeting NEK7 for downregulation, thereby suppressing NLRP3 inflammasome activation and subsequent cytokine secretion.

## 3. Discussion

The activation of the NLRP3 inflammasome requires two distinct signals. LPS acts as a pathogen-associated molecular pattern (PAMP) to prime NLRP3 inflammasome activation, whereas ATP functions as a damage-associated molecular pattern (DAMP) that triggers inflammasome assembly and activation [22]. Recent evidence indicates that ROS are critical to signal transduction, and that a number of compounds, for example, ATP, serve as triggers for NLRP3 inflammasome activation through elevated ROS [23].

LPS are a kind of endotoxin, which prompts the synthesis and subsequent release of pro-inflammatory cytokines. LPS released during bacterial infection are recognized and activated by macrophages, the primary innate immune response of the host. It was found that the activity of 3D4/21 cells was inhibited by 1 μg/mL of LPS. Therefore, LPS of 1 μg/mL demonstrated a low cell survival in all subsequent trials. ATP is one of the organic compounds in which phosphate binding is formed. ATP is a known activator of the NLRP3 inflammasome. For example, elevated hydrostatic-pressure-induced ATP release could activate the NLRP3 inflammasome through engagement of the P2X4 receptor in rat urothelial cells [24]. The classic mechanism by which NLRP3 is activated is through LPS and ATP. LPS and ATP treatment resulted in a significant increase in the protein levels of NLRP3 and ASC. LPS and ATP also caused effective aggregation of ASC punctate specks, and previous studies have proved that stimulation with a combination of 1 μg/mL LPS and 5 mM ATP activates the NLRP3 inflammasome [25]. Therefore, in the present study, co-stimulation of 3D4/21 cells using 1 μg/mL LPS and 5 mM ATP was used to establish an inflammation model.

Once activated, the NLRP3 inflammasome cleaves pro-caspase-1 into its active form, which in turn processes pro-IL-1β and pro-IL-18 into their mature, secreted forms. This cascade is governed by a complex molecular framework encompassing multiple signaling pathways. A key mechanism entails PAMPs and DAMPs triggering intracellular ROS accumulation via ROS-dependent signaling cascades, which in turn promote NLRP3 inflammasome assembly and activation [26]. ROS function as critical signaling that initiate host inflammatory responses and regulate cellular signal transduction [27]. Functioning as a cornerstone of innate immunity, the NLRP3 inflammasome orchestrates the activation of caspase-1 and the subsequent secretion of the pro-inflammatory cytokines IL-1β and IL-18. This response is triggered upon detection of microbial pathogens or signs of cellular damage [28]. In our experimental model, LPS/ATP stimulation markedly upregulated the generation and secretion of inflammatory cytokines IL-8 and IL-1β from 3D4/21 cells—a process driven by intracellular ROS generation. According to the literature, Eucommia polysaccharides can exert anti-inflammatory and anti-oxidant effects by reducing ROS levels and inhibiting TLR4/NF-κB pathway activation to reduce hepatic ischemia-reperfusion injury [29]. The sulfated polysaccharide, luteolin, isolated from *Ascophyllum nodosum* resulted in reduced levels of NO and ROS in LPS-stimulated RAW264.7 cells [30]. Astragalus polysaccharides can attenuate Cd-induced autophagic damage in chick embryo fibroblasts by reducing the production of ROS [31]. Therefore, to explore how PGPS_t_ affects NLRP3 inflammasome activation under LPS/ATP stimulation, we first examined the effect of PGPS_t_ on ROS production. The study revealed that LPS/ATP co-stimulation robustly enhanced intracellular ROS levels and activated the NLRP3 inflammasome in 3D4/21 cells, causing the processing and subsequent secretion of both IL-1β and IL-18. Conversely, PGPS_t_ treatment significantly suppressed the LPS/ATP-triggered response, encompassing both ROS generation and NLRP3 inflammasome activation. Hence, PGPSt can partially suppress NLRP3 inflammasome activation by blocking a key ROS-dependent step.

NEK7 is an essential component for NLRP3 inflammasome activation and is recruited prior to its activation. NEK7 plays a critical role in the oligomerization of NLRP3, promoting inflammasome activation when cells are exposed to danger signals such as nigericin, ATP, and gramicidin [32]. It has been established that stimulation with LPS and ATP induces NEK7-dependent NLRP3 inflammasome activation, involving a series of events: NLRP3 assembly, ASC speck formation, caspase-1 activation, and IL-1β secretion. Inhibition of NEK7 counteracts these inflammatory responses, leading to reduced TBI severity along with diminished inflammatory cytokine release in model systems [33]. Although NEK7 holds promise as a therapeutic target for disorders involving the NLRP3 inflammasome, highly selective inhibitors of this protein remain scarce. Recently, oridonin has been shown to covalently bind to Cys279 of NLRP3 and block NEK7–NLRP3 interactions, leading to the subsequent NLRP3 inflammasome assembly and activation [22]. Furthermore, the latest study showed that berberine directly targets NEK7 to inhibit the interaction between NLRP3 and NEK7 [34]. These studies suggest that inhibition of NEK7–NLRP3 interactions may be an excellent strategy to inhibit NLRP3 inflammasome activation. After using the MCC950 inhibitor, accordingly, the interaction between NLRP3 and NEK7 was inhibited by PGPS_t_. In addition, the absence of NEK7 may lead to specific blockage of NLRP3 inflammasome activation. Herein, the application of NEK7 siRNA transfection, aimed at blocking NLRP3 inflammasome activation and the generation of inflammatory factors, was followed by the finding that PGPS_t_ mimicked the outcome of NEK7 silencing. Therefore, PGPS_t_ can alleviate LPS/ATP-induced cellular inflammatory response by downregulating NEK7 expression, and NEK7 may be a potential target for the development of new anti-inflammation strategies of PGPS_t_. The docking analysis suggests a potential interaction between D-Glucose and NEK7/NLRP3; however, this observation should be interpreted cautiously, as D-Glucose represents only a single monosaccharide component of PGPS_t_. Further biochemical and structural studies are required to determine whether the intact polysaccharide directly interacts with the NEK7–NLRP3 complex.

NEK7 binds to the leucine-rich repeat area of NLRP3 in a kinase-independent manner in the downstream pathway of mitochondrial ROS [35]. NEK7 appears to participate in the ROS-mediated activation of the NLRP3 inflammasome. ROS may be located upstream of the NEK7 action site. Furthermore, the activation of NEK7 and its subsequent engagement with NLRP3 may depend on ROS. Previous research indicated that UA activation of ROS in NRK-52E cells may be associated with the NEK7/NLRP3 signaling pathway [36]. The results indicated that NEK7, NLRP3, ASC, and caspase-1 were upregulated by LPS/ATP-induced ROS. Inhibition of NEK7/NLRP3 activation was also observed by NAC; this suggests that NAC counteracts LPS/ATP-induced inflammation by targeting the NEK7/NLRP3 signaling axis.

Several limitations of this study should be acknowledged. First, all experiments were conducted in vitro using porcine alveolar macrophages (3D4/21 cells), and further validation in animal models of inflammatory disease is required to confirm the anti-inflammatory efficacy of PGPSt in vivo. Second, long-term biological effects and systematic toxicity/safety evaluations were not performed, warranting future investigation. Third, the proposed interaction between PGPSt-derived sugar units and the NEK7–NLRP3 complex is currently supported only by computational docking analyses and requires biochemical validation (e.g., co-immunoprecipitation or pull-down assays). Finally, pharmacokinetic characteristics, molecular stability, and long-term therapeutic efficacy remain to be investigated in future translational studies.

## 4. Materials and Methods

### 4.1. Materials and Chemicals

*Platycodon grandiflorus* polysaccharide (PGPS_t_) was extracted and purified according to established protocols involving hot-water extraction, ethanol precipitation, deproteinization, and dialysis [1,37]. These procedures ensured a high-purity preparation with minimal protein and nucleic acid contamination, while endotoxin levels were strictly controlled during processing to avoid confounding immunological effects.

The physicochemical properties of PGPS_t_ have been well documented in our previous studies. PGPS_t_ possesses a weight-average molecular weight (Mw) ranging from 2.05 × 10^3^ to 2.67 × 10^5^ Da. It is primarily composed of glucose (55.397%) and mannose (22.305%), with a backbone structure consisting of repeating (1 → 3)-β-D-Glcp-(1 → 6)-β-D-Glcp linkages, as confirmed by NMR spectroscopy. Furthermore, previous quality control analyses indicated the absence of protein and nucleic acid impurities and confirmed the presence of a triple-helix conformation.

Gibco provided the modified RPMI-1640 medium. Every Green provided the bovine serum albumin. LPS (L2880) and ATP (A2383) were purchased from Sigma (St. Louis, MO, USA). The Hoechst 33342 nuclear staining kit (C1026) was obtained from Beyotime Biotechnology (Shanghai, China).

The following method was used to obtain primary antibodies: anti-tubulin (66031), anti-NLRP3 (68102), anti-ASC (67494), and anti-caspase-1 (81482) from Proteintech (Rosemont, IL, USA); anti-NEK7 (bs-7758R) from Bioss (Woburn, MA, USA); anti-IL-1β (RAA563Hu21) from Cloud-Clone Corp (Wuhan, China). Corresponding secondary antibodies were Goat Anti-Mouse/Rabbit IgG (ABclonal Biotechnology, Wuhan, China) and Alexa Fluor 488-conjugated Donkey Anti-Rabbit IgG (Abcam, Shanghai, China).

The concentrations of IL-1β and IL-18 in the cell culture supernatants were quantified using porcine-specific ELISA kits (RayBiotech, Norcross, GA, USA).

### 4.2. Cell Culture

iCell Bioscience Inc. (Shanghai, China) provided the porcine alveolar macrophages (3D4/21 cells), which were grown in RPMI-1640 with 10% bovine serum albumin.

### 4.3. Experimental Treatments

RPMI-1640 medium containing 10% fetal bovine serum and antibiotics was used to cultivate 3D4/21 cells. The cells were subjected to various treatments for a total of 5 h at 37 °C. The experimental groups were designed as follows: (1) control group: cultured in 1640; (2) LPS/ATP group: treated with 1 μg/mL LPS and 5 mM ATP; (3) PGPSt+LPS/ATP group: treated with 200 μg/mL PGPS_t_ and 1 μg/mL LPS along with LPS/ATP; (4) NAC+LPS/ATP group: treated with 5 mM NAC and LPS/ATP; (5) MCC950+LPS/ATP group: treated with 5 mM MCC950 and LPS/ATP; (6) siNEK7+LPS/ATP group: transfected with siNEK7 and then treated with LPS/ATP.

### 4.4. Assay for Cell Viability

The Cell Counting Kit (CCK-8) assay was used to assess cell viability. We incubated the cells with CCK-8 solution in 5% CO2 at a temperature of 37 °C for a duration of 1 h. We then measured the absorbance of the samples at a wavelength of 450 nm.

### 4.5. Western Blot Analysis

After the treatments with LPS/ATP, PGPS_t_, or specific inhibitors, 6-well plate-cultured cells were harvested for total protein extraction. SDS-PAGE was used to separate the isolated proteins before they were transferred onto PVDF membranes. Next, the membranes were incubated with specific primary antibodies against the proteins NLRP3, NEK7, ASC, IL-1β, caspase-1, and tubulin. Protein band signals were visualized via enhanced chemiluminescence (ECL) detection, with band intensities quantified using computer-based image analysis software.

### 4.6. Immunofluorescent Staining and ROS Measurement

Cells were fixed specifically for immunofluorescence analysis using 4% paraformaldehyde (*w*/*v*). The fixed cells were immunostained with specified primary antibodies (anti-tubulin, anti-ASC, anti-NEK7) and a secondary antibody conjugated to Alexa Fluor 488, with nuclei labeled by Hoechst 33342. For intracellular ROS measurement, live cells were incubated with 10 μM DCFH-DA for 30 min before fixation, after which fluorescence images were captured using a confocal laser scanning microscope.

### 4.7. Flow Cytometry Analysis

Intracellular ROS levels were determined by flow cytometry. Following respective treatments, cells were harvested and stained using the Reactive Oxygen Species Assay Kit (Beyotime, China), strictly based on the guidelines provided by the manufacturer. Subsequent information acquisition and analysis were performed on a commercial flow cytometer instrument.

### 4.8. ELISA Analysis

Concentrations of IL-1β and IL-18 in cell-free supernatants were quantified via ELISA kits, in strict accordance with the manufacturers’ protocols. Following completion of all assay procedures, we recorded absorbance readings at 450 nm for each well with a microplate reader. Actual cytokine concentrations (pg/mL) were then determined by interpolating the measured absorbance values against the standard curve generated for each assay.

### 4.9. Quantitative Polymerase Chain Reaction in Real-Time Fluorescence

We isolated the total RNA from the samples using RNAiso Plus reagent (Accurate Biology, Changsha, China) for subsequent molecular analysis. RNA samples were then treated with DNase to remove genomic DNA contamination, and reverse-transcribed into complementary DNA (cDNA) using a commercial kit (Accurate Biology, Changsha, China), in strict accordance with the method used by the manufacturer. For quantitative PCR analysis, amplification was conducted with SYBR Green Master Mix (Applied Biosystems, Shanghai, China) and gene-specific primer pairs (Table 1) on a Roche LightCycler^®^ 96 real-time PCR system (Roche Diagnostics International AG, Rotkreuz, Switzerland).

### 4.10. Statistical Analysis

All data are expressed as the mean ± SD from at least three independent experiments. Statistical analyses were performed using SPSS 27.0 software (IBM, Armonk, NY, USA). Student’s *t*-test was used to compare differences between two groups, and one-way analysis of variance (ANOVA) was applied for multiple-group comparisons. For the purpose of statistical analysis, any *p* value less than 0.05 was deemed statistically significantly different.

## 5. Conclusions

In summary, this study establishes PGPS_t_ as a potent NLRP3 inflammasome inhibitor. We delineate a dual mechanism whereby PGPS_t_ scavenges ROS and directly disrupts the NLRP3–NEK7 interaction, the latter being supported by molecular docking and validated by NEK7 silencing. Our work thus nominates PGPS_t_ as a promising therapeutic agent and highlights NEK7 as a novel target for treating NLRP3-driven inflammatory diseases.

## Figures and Tables

**Table 1:** Primer sequences (*Sus scrofa*) for real-time PCR analysis.

Gene Name	Primer	Sequence (5′–3′)
IL-1β	Forward: Reverse:	CTCTCCAGCCAGTCTTCATTG GGGCCATCAGCCTCAAATAAC
IL-18	Forward: Reverse:	CTGCTGAACCGGAAGACAA ACACGGCTTGATGTCCCT
GAPDH	Forward: Reverse:	CACTGGTGTCTTCACGACCAT TTCACGCCCATCACAAACA

## Data Availability

The original contributions presented in this study are included in the article. Further inquiries may be directed to the corresponding authors.

## Abbreviations

The following abbreviations are used in this manuscript:

ASC	Apoptosis-associated speck-like protein containing a CARD
Caspase-1	Cysteine-dependent aspirate protease 1
NAC	N-acetyl-L-cysteine
NEK7	NIMA (never in mitosis gene a)-related kinase 7
NLRP3	Nucleotide-binding oligomerization domain (nod)-like receptor family pyrin-domain-containing 3
PGPS_t_	Total platycodon grandifloras polysaccharide
3D4/21 cells	Porcine alveolar macrophages

## References

[1] Zheng P., Fan W., Wang S., Hao P., Wang Y., Wan H., Hao Z., Liu J., Zhao X. (2017). Characterization of polysaccharides extracted from *Platycodon grandiflorus* (Jacq.) A. DC. affecting activation of chicken peritoneal macrophages. Int. J. Biol. Macromol..

[2] Guo X., Zhao X., Li L., Jiang M., Zhou A., Gao Y., Zheng P., Liu J., Zhao X. (2024). *Platycodon grandiflorus* polysaccharide inhibits the inflammatory response of 3D4/21 cells infected with PCV2. Microb. Pathog..

[3] Hao J., Song Y., Tian B., Qi C., Li L., Wang L., Xing Y., Zhao X., Liu J. (2020). *Platycodon grandifloras* polysaccharides inhibit mitophagy injury induced by Cr (VI) in DF-1 cells. Ecotoxicol. Environ. Saf..

[4] Qi C., Li L., Cheng G., Xiao B., Xing Y., Zhao X., Liu J. (2021). Platycodon grandiflorus Polysaccharide with Anti-Apoptosis, Anti-Oxidant and Anti-Inflammatory Activity Against LPS/D-GalN Induced Acute Liver Injury in Mice. J. Polym. Environ..

[5] Gao X., Cai S., Li X., Wu G. (2025). Sepsis-induced immunosuppression: Mechanisms, biomarkers and immunotherapy. Front. Immunol..

[6] Bai X., Guo Y.-R., Zhao Z.-M., Li X.-Y., Dai D.-Q., Zhang J.-K., Li Y.-S., Zhang C.-D. (2025). Macrophage polarization in cancer and beyond: From inflammatory signaling pathways to potential therapeutic strategies. Cancer Lett..

[7] Zhang T., Ding S., Wang R. (2021). Research Progress of Mitochondrial Mechanism in NLRP3 Inflammasome Activation and Exercise Regulation of NLRP3 Inflammasome. Int. J. Mol. Sci..

[8] Meier D.T., de Paula Souza J., Donath M.Y. (2025). Targeting the NLRP3 inflammasome-IL-1beta pathway in type 2 diabetes and obesity. Diabetologia.

[9] Dominic A., Le N.T., Takahashi M. (2022). Loop Between NLRP3 Inflammasome and Reactive Oxygen Species. Antioxid. Redox Signal..

[10] Peláez-Vico M.Á., Fichman Y., Zandalinas S.I., Foyer C.H., Mittler R. (2024). ROS are universal cell-to-cell stress signals. Curr. Opin. Plant Biol..

[11] Ni X., Wang Q., Ning Y., Liu J., Su Q., Lv S., Feng Y., Yang S., Yuan R., Gao H. (2025). Anemoside B4 targets NEK7 to inhibit NLRP3 inflammasome activation and alleviate MSU-induced acute gouty arthritis by modulating the NF-kappaB signaling pathway. Phytomedicine.

[12] Sehnert B., Burkhardt H., Dübel S., Voll R.E. (2020). Cell-Type Targeted NF-kappaB Inhibition for the Treatment of Inflammatory Diseases. Cells.

[13] Panchal N.K., Evan Prince S. (2023). The NEK family of serine/threonine kinases as a biomarker for cancer. Clin. Exp. Med..

[14] Aziz M., Ejaz S.A., Zargar S., Akhtar N., Aborode A.T., Wani T., Batiha G.E., Siddique F., Alqarni M., Akintola A.A. (2022). Deep Learning and Structure-Based Virtual Screening for Drug Discovery against NEK7: A Novel Target for the Treatment of Cancer. Molecules.

[15] Picco C.M., Bastida G.A., Tarres Q., Regenhardt S.A., Delgado-Aguilar M. (2025). Extracting value from spent yerba mate residue: Lignocellulose-reinforced poly-l-lactic acid composites for fused deposition modeling. Int. J. Biol. Macromol..

[16] Wang X., Li J., Yu B., Gong H., Li Z., Bai Y. (2026). Nimbolide inhibits NLRP3 inflammasome activation via blocking NEK7-NLRP3 interaction and alleviates sepsis-induced lung injury. Int. Immunopharmacol..

[17] Li L., Xu H., Wang Y., Zhang Y., Ye R., Li W., Yang J., Wu J., Li J., Jin E. (2024). From inflammation to pyroptosis: Understanding the consequences of cadmium exposure in chicken liver cells. Ecotoxicol. Environ. Saf..

[18] Zhao W., Liu Y., Hu Y., Zhang G. (2025). SOX4 accelerates intervertebral disc degeneration via EZH2/NRF2 pathway in response to mitochondrial ROS-dependent NLRP3 inflammasome activation in nucleus pulposus cells. J. Transl. Med..

[19] Gupta S., Cassel S.L., Sutterwala F.S., Dagvadorj J. (2024). Regulation of the NLRP3 inflammasome by autophagy and mitophagy. Immunol. Rev..

[20] Ren K., Li X. (2025). MCC950 targets the ROS-NEK7-NLRP3 axis to improve type 2 diabetic retinopathy. Sci. Rep..

[21] Lv Q., Yang H., Xie Y., Huang X., Yan Z., Lv Y., Cui Y., Hu L., Qiao H. (2025). *Prunus mume* derived extracellular vesicle-like particles alleviate experimental colitis via disrupting NEK7-NLRP3 interaction and inflammasome activation. J. Nanobiotechnol..

[22] Lamichhane P.P., Aditi, Neil B.H., Kilgore P.B., Torres A.G., Chopra A.K., Samir P. (2025). Stress granule component TIA-1 is a negative regulator of the non-canonical NLRP3 inflammasome. bioRxiv.

[23] Li P., Li S., Wang L., Li H., Wang Y., Liu H., Wang X., Zhu X., Liu Z., Ye F. (2023). Mitochondrial dysfunction in hearing loss: Oxidative stress, autophagy and NLRP3 inflammasome. Front. Cell Dev. Biol..

[24] Oda K., Miyamoto S., Kodera R., Wada J., Shikata K. (2022). Suramin prevents the development of diabetic kidney disease by inhibiting NLRP3 inflammasome activation in KK-Ay mice. J. Diabetes Investig..

[25] Xu S., Zhou Q., Fan C., Zhao H., Wang Y., Qiu X., Yang K., Ji Q. (2019). Doxycycline inhibits NAcht Leucine-rich repeat Protein 3 inflammasome activation and interleukin-1beta production induced by Porphyromonas gingivalis-lipopolysaccharide and adenosine triphosphate in human gingival fibroblasts. Arch. Oral Biol..

[26] Duan J.-X., Jiang H.-L., Guan X.-X., Zhang C.-Y., Zhong W.-J., Zu C., Tao J.-H., Yang J.-T., Liu Y.-B., Zhou Y. (2021). Extracellular citrate serves as a DAMP to activate macrophages and promote LPS-induced lung injury in mice. Int. Immunopharmacol..

[27] Tang S.P., Mao X.L., Chen Y.H., Yan L.L., Ye L.P., Li S.W. (2022). Reactive Oxygen Species Induce Fatty Liver and Ischemia-Reperfusion Injury by Promoting Inflammation and Cell Death. Front. Immunol..

[28] Sharma B.R., Wang Y., Choudhury S.M., Abdelaal H.M., Kanneganti T.D. (2025). Innate immune sensor NLRP3 drives PANoptosome formation and PANoptosis. J. Immunol..

[29] Gao W., Feng Z., Zhang S., Wu B., Geng X., Fan G., Duan Y., Li K., Liu K., Peng C. (2020). Anti-Inflammatory and Antioxidant Effect of Eucommia ulmoides Polysaccharide in Hepatic Ischemia-Reperfusion Injury by Regulating ROS and the TLR-4-NF-kappaB Pathway. Biomed. Res. Int..

[30] Liang Y., Zha S., Tentaku M., Okimura T., Jiang Z., Ueno M., Hirasaka K., Yamaguchi K., Oda T. (2021). Suppressive effects of sulfated polysaccharide ascophyllan isolated from *Ascophyllum nodosum* on the production of NO and ROS in LPS-stimulated RAW264.7 cells. Biosci. Biotechnol. Biochem..

[31] Shen X., Tang Z., Bai Y., Wan M., Yu M., Chen J., Li G., Zhang R., Ge M. (2022). Astragalus Polysaccharide Protects Against Cadmium-Induced Autophagy Injury Through Reactive Oxygen Species (ROS) Pathway in Chicken Embryo Fibroblast. Biol. Trace Elem. Res..

[32] Shi X., Dong J., Yang Y., He Y., Li H., Du T., Yang B., Yang C., Zhang P., Xin T. (2026). Methylglyoxal-modification of NLRP3 interrupts NLRP3-NEK7 interaction diminishing inflammasome activation and neuroinflammation. J. Neuroinflam..

[33] Kelley N., He Y. (2023). Assessment of NLRP3 Inflammasome Activation and NLRP3-NEK7 Complex Assembly. Methods Mol. Biol..

[34] Zeng Q., Deng H., Li Y., Fan T., Liu Y., Tang S., Wei W., Liu X., Guo X., Jiang J. (2020). Berberine Directly Targets the NEK7 Protein to Block the NEK7–NLRP3 Interaction and Exert Anti-inflammatory Activity. J. Med. Chem..

[35] Caseley E.A., Lara-Reyna S., Poulter J.A., Topping J., Carter C., Nadat F., Spickett G.P., Savic S., McDermott M.F. (2021). An Atypical Autoinflammatory Disease Due to an LRR Domain NLRP3 Mutation Enhancing Binding to NEK7. J. Clin. Immunol..

[36] Li D., Wang L., Ou J., Wang C., Zhou J., Lu L., Wu Y., Guo J. (2021). Reactive oxygen species induced by uric acid promote NRK-52E cell apoptosis through the NEK7-NLRP3 signaling pathway. Mol. Med. Rep..

[37] Cheng G., Zhang S., Lv M., Qi C., Fan R., Guo X., Liu J., Zhao X. (2022). The surface morphology of Platycodon grandiflorus polysaccharide and its anti-apoptotic effect by targeting autophagy. Phytomedicine.

